# Constructing chimeric mouse islets to study alpha- and delta-cell influence on beta-cell feature

**DOI:** 10.1016/j.molmet.2025.102245

**Published:** 2025-09-01

**Authors:** Alexis Fouque, Masaya Oshima, Nina Mode, Romain Ducellier, Delphine Thibaut, Florence Gbahou, Latif Rachdi, Over Cabrera, Raphaël Scharfmann

**Affiliations:** 1 Université de Paris, Institut Cochin, INSERM U1016, CNRS, UMR8104, Paris, France; 2 Lilly Research Laboratories, Eli Lilly and Company, Indianapolis, IN, USA

**Keywords:** Pancreas, Islets, Endocrine cells, Chimeric clusters, Insulin secretion

## Abstract

**Objectives:**

This study aimed to evaluate the role of alpha- and delta-cell signals on beta-cells within pancreatic mouse islets. Specifically, we investigated how these signals regulate glucose sensitivity, gene expression and function in beta-cells.

**Methods:**

We first implemented our previous protocol to FACS purify alpha-, beta-, and delta-cells by adding CD81 as a positive marker for alpha-cells. We next developed an approach to reaggregate these sorted cell populations, creating chimeric islets with different proportions of each endocrine cell type. We used these chimeric islets to study the effect of alpha- and delta-cells on glucose sensitivity, gene expression and function in beta-cells.

**Results:**

We generated chimeric islets containing either all three endocrine cell types, alpha- + beta-cells or only beta-cells. We demonstrate that beta-cell glucose sensitivity and identity are independent of signals from alpha- and delta-cells. We identified a subset of genes including Pro-dynorphin, Fumarate hydratase and Txnip whose expression in beta-cells depends on alpha-cells signals acting through the glucagon- and glucagon-like peptide receptors. Finally, we demonstrated that in mouse beta-cell, KCl-mediated insulin secretion relies on an activation of the glucagon-receptor, while glucose-stimulated insulin secretion depends on glucagon-like peptide receptor activation.

**Conclusions:**

We developed an innovative and easy-to-use model to reconstruct chimeric islets containing different frequencies of alpha-, beta- and delta-cells. Through this approach, we provide new insights into the complex regulatory mechanisms governing the role of alpha and delta cells on beta-cell features within islets.

## Highlights

• An innovative method to produce chimeric mouse islet clusters.
• Alpha- and delta-cell are not essential for beta-cell identity or glucose sensitivity.
• Alpha-cells are essential for optimal beta-cell function.
• In beta cells, a subset of new genes is regulated by GCGR and GLP1R signaling.
• GLP1R and GCGR signaling is necessary for glucose- and KCl-induced insulin secretion.

## 1 Introduction

The pancreas is a mixed gland primarily composed of exocrine tissue, which secretes digestive enzymes into the digestive tract, and an endocrine component organized into small clusters known as islets of Langerhans, constituting approximately 1% of the pancreatic mass. Each adult islet contains an average of 1,500 cells, including beta-, alpha- and delta-cells, which produce and secrete insulin (INS), glucagon (GCG), and somatostatin (SST) respectively [1]. The destruction of insulin-producing beta-cells or the defective insulin secretion give rise to type 1 and type 2 diabetes mellitus, respectively. These chronic metabolic disorders are characterized by the dysregulation of glucose homeostasis. The pathophysiology of diabetes has been extensively studied and beta-cell biology is now described in great details. Glucose is taken up by beta-cells, metabolized and generates an increase in the intracellular ATP:ADP ratio that drives the closure of ATP-sensitive potassium (K_ATP_) channels. This causes membrane depolarization, leading to the activation of voltage-gated calcium channels, which increases intracellular calcium level and initiates insulin secretion [2]. This secretory process is enhanced by signals mediated by insulinotropic Gαs coupled G Protein Coupled Receptors (GPCRs) that increase cAMP levels. For example, this occurs through GCG-, Glucagon-like Peptide 1- (GLP1) and Glucose-dependent insulinotropic polypeptide (GIP)-Receptors, all of which are expressed at the beta-cell surface [2]. In parallel, SST secreted by delta-cells, by acting through its receptors expressed on beta-cells, decreases cAMP levels and insulin secretion [3,4]. Drugs targeting beta-cell secretion are used to treat patients suffering of type 2 diabetes. They increase insulin secretion by closing the K_ATP_ channels (sulfonylureas) or by increasing intracellular cAMP (GLP1R agonists). However, many aspects of pancreatic islet function remain to be further understood. Specifically, more needs to be learned about the role of signals from alpha- and delta-cells on beta-cells within the islets.

Past studies on cell interactions within rodent islets relied on several approaches: the use of pharmacological activators and inhibitors of specific pathways, such as GCGR or GLP1R [5], genetic deletion of specific receptors from beta-cells [3,6] or specific destruction of alpha- and delta-cells [7]. These methods have provided valuable insights but have some limitations: they rely on the use of activators at concentrations difficult to compare to physiological levels within islets or involve complex genetic manipulations that are challenging to implement in diverse experimental systems. Here, we aimed to develop a simple and robust experimental model to study how signals from alpha- and delta-cells regulate beta-cell stability and function.

In previous studies [8,9], we developed a FACS strategy utilizing the cell surface markers CD24, CD49f, and CD71 to efficiently isolate live alpha-, beta-, and delta-cell populations from any mouse pancreatic islet strain. Building upon this foundation, we first implemented this FACS protocol by adding the alpha-cell enriched cell surface marker CD81 [10] to further increase the purity of sorted islet cell populations. We next developed a culture model where sorted live cells can be reaggregated and kept functional, giving rise to reconstructed islets, with controlled proportion of alpha-, beta-, and delta-cell on demand. These chimeric islets serve as a robust system for dissecting cell–cell interactions within the islets, allowing us to investigate the influence of alpha- and delta-cells on beta-cell glucose sensitivity, gene expression and function.

By using this new and easy-to-use model of chimeric islets, (i) we demonstrate that beta-cell sensitivity to glucose is independent of the presence of alpha- and delta-cell. It is also the case for the expression of beta cell identity genes; (ii) we identify a new subset of beta-cell genes positively or negatively regulated by signaling from alpha-cells through the GCGR and the GLP1R; (iii) we show that beta-cell function in mouse islets depends on alpha-cell signals, with GCG-mediated signaling influencing potassium chloride (KCl)-induced beta-cell activity and GLP1R signaling modulating glucose-induced insulin secretion.

These findings provide new insights into the complex regulatory mechanisms governing beta-cell function and may inform future therapeutic strategies for diabetes.

## 2 Materials and methods

### 2.1 Animals procedure

Animal studies were certified by the Direction Départementale de la Protection des Populations for the French Ministry of Research, Health, and Agriculture (Paris), under agreement number A75-13-19, in accordance with approved guidelines of French and European legislation. Twelve-week-old C57BL/6JRj male mice (Janvier Labs, Saint Berthevin, France) were housed under a 12-hours light/dark cycle with *ad libitum* access to food and water. Animals were killed by cervical dislocation.

### 2.2 Mouse pancreatic islets isolation

Mouse islet isolation was performed as described in [8]. Type V collagenase solution (Sigma–Aldrich, #C9263) was injected into the pancreas through the bile duct. The inflated pancreas was digested for 20 min at 37 °C. Digested pancreas was re-suspended in HBSS^+/+^ (Hank's Balanced Salt Solution; Life Technologies, #14025–050). Islets were handpicked and cultured overnight at 37 °C in mouse islets culture medium: RPMI 1640 (Gibco, #14025–050) supplemented with 10% Fetal Calf Serum (FCS, Eurobio, #CVFSVF00-01) and penicillin/streptomycin (Pen/Strep, Gibco, #15140–122).

### 2.3 Flow cytometry

Islet clusters were dissociated in single-cell suspension using Accutase (StemCell Technologies, #07920). Cells were stained for 15 min at 4 °C in the dark, with antibodies in FACS medium (HBSS^−/-^ + 10%FCS + Pen/Strep). Antibodies used for sorting were: CD45 (1:100, BioLegend, #103132, RRID AB_893340), TER119 (1:100, BioLegend, #116228, RRID AB_893636), CD31 (1:100, BioLegend, #102522, AB_2566761), CD24 (1:100, BioLegend, #101840, RRID AB_2650876), CD49f (1:200, BioLegend, #313612, RRID AB_893373), CD71 (1:100, BioLegend, #113806, RRID AB_313567) and CD81 (1:100, BD Bioscience, #740060, RRID AB_2739825). Stained cells were resuspended in FACS medium supplemented with 1:4,000 propidium iodide (Sigma–Aldrich, #P4864) to assess viability. Sorting has been performed on a FACSAria III (BD Biosciences). Data were analyzed using FlowJo™ software 10.7.1 (RRID: 008520).

### 2.4 RT-qPCR

RNA was extracted using the RNeasy Micro Kit (Qiagen, #74004) and reverse-transcribed into cDNA using the Maxima First Strand cDNA Synthesis Kit (ThermoFisher Scientific, #K1642). RT-qPCR was performed using the Power SYBR™ Green PCR Master Mix (ThermoFisher Scientific, #4367659) on a QuantStudio™ 3 System (Thermo Fisher Scientific). Serial dilution validated primer sequences (Eurofins France) are listed in (Suppl Table S1). Gene expression levels were quantified using the 2^−ΔCt^ method, with the *Cyclophilin-A* (*Ppia*) gene serving as the housekeeping gene. Fold-change was calculated using the 2ˆ^−ΔΔCt^ method, relative to the control group.

### 2.5 Reaggregation and cell culture

A 2% agarose (#A9539 Sigma Aldrich) 0.9% NaCl solution was poured into silicone-micro cast (Sigma Aldrich, #Z764051). After solidification at RT, agarose molds were kept in NaCl solution at 4 °C until use. Prior to cell seeding, molds were rinsed for 10 min twice at 37 °C with islet medium. Dispersed islets cells or sorted cells were seeded in agarose molds and incubated at 37 °C, 5%CO_2_. Pseudo-islets were cultured in islet medium supplemented or not with GCG (GlucaGen; Novo Nordisk), Ex4 (Bachem, #4027457), Ex9 (Bachem, #4017799), IUB288 (provided by Eli Lilly) or SST (Phoenix, #060–21).

### 2.6 Immunofluorescences and quantification

Islets and pseudo-islets were fixed in 3.7% formaldehyde solution (Merck, #104003) containing Light Green (Reactifs RAL, #42095), pre-embedded in agarose (Sigma–Aldrich, #A4018), dehydrated and embedded in paraffin (Histolab, #HistoWax). They were sliced (4 μm-thick sections), deparaffinized and boiled in citrate-based antigen retrieval solution (BioGenex, #HK086–9K). They were permeabilized in TBS (Sigma, #T6664) supplemented with 3% BSA (Sigma, #A7906) and 0.3% Triton X-100 (Sigma #T8787) for 30 min. Slides were next incubated with primary antibodies diluted in the blocking solution overnight at 4 °C. Secondary antibodies and Hoechst 33342 dye (Invitrogen, #H3570) were diluted in the blocking solution for 3h at RT. The antibodies used are: insulin (1:2,000, Sigma–Aldrich, #I2018), glucagon (1:1,000, Immunostar, #20279), somatostatin (1:1,000, Abcam, #ab108456), P-RPS6 (1:500, Cell Signaling, #5364L), anti-mouse AF594 (1:400, Jackson ImmunoResearch, #115-585-003), anti-rabbit AF488 (1:400, Invitrogen, #A11034). Images were captured using a Leica, #DM4000B fluorescence microscope and Wasabi 1.4 software (Hamamatsu Photonics Germany GmbH). Quantifications were performed using the ImageJ software (U. S. National Institutes of Health, RRID: 003070).

### 2.7 Western Blot

Mouse pancreatic islets or pseudo-islets were cultured overnight in RPMI 1640 containing 2 mM glucose, supplemented with 2% FCS and penicillin/streptomycin. The following day, islets were stimulated for 15min at either 2.8 mM or 16.7 mM glucose. For experiments related to protein translation, puromycin (ThermoFischer Scientific; #A1113803) was added during the last 15 min at 10 μg/ml. Samples were lysed in RIPA buffer (Sigma–Aldrich, #R0278) supplemented with protease and phosphatase inhibitors (Roche #04693159001 and #04906837001). Equal amounts of proteins in Laemmli buffer (Bio-Rad, #1610747) supplemented with 2-β-Mercaptoethanol (Sigma, #M3148) were loaded onto 4–12% Bis-Tris polyacrylamide gels and transferred onto PVDF membrane using a semi-dry transfer system (iBLOT 2 Dry Blotting System, Thermo Fisher Scientific). Membrane was blocked in TBS/0.1% Tween/5% milk. Membrane was then incubated with the primary antibody in the same buffer overnight at 4 °C, washed 3 times and incubated with an HRP-conjugated secondary antibody for 1h at RT. Proteins were detected using the ECL Prime (Cytiva, #RPN2236) and visualized with an iBright 1500 (Thermo Fisher Scientific). Quantification was performed using ImageJ software (U. S. National Institutes of Health, RRID: 003070) and normalized on α-tubulin. Antibodies used are the following: P-RPS6 (1:1,000, Cell Signaling, #5364L), RPS6 (1:1,000, Cell Signaling, #2217S), INS (1:1,000, Cell Signaling, #3014T), puromycin (1:1,000, Sigma–Aldrich, #MABE343), tubulin (1:1,000, Sigma–Aldrich, #T9026), anti-mouse HRP (1:2,000, Cell Signaling, #7076S) and anti-rabbit HRP (1:2,000, Cell Signaling, #7074S).

### 2.8 INS immunoprecipitation

Proteins (80 μg) were diluted in RIPA buffer and incubated with 1 μg of anti-insulin antibody (Cell Signaling #8138) at 4 °C overnight under agitation. Agarose beads (Pierce™ Protein A/G Agarose, Thermofisher Scientific, #20421) were washed in RIPA buffer overnight at 4 °C under agitation, added to protein samples and incubated at RT under agitation for 2–3h. Immunoprecipitated samples were eluted by resuspending the beads in Laemmli buffer supplemented with 2-β-mercaptoethanol, and boiled for 5–10min. Beads were removed by centrifugation, and the protein samples were stored at −20 °C until Western Blot assay.

### 2.9 Next-generation sequencing

Total RNAs were prepared from sorted beta-cells and their quality was assessed using the Agilent 2100 Bioanalyzer. Libraries were prepared from 5 ng of total RNA from each sample using the *Ovation solo RNA-seq kit* (NuGen, #0501–96) followed by ribosomal RNA depletion using the *AnyDeplete* technology (NuGen). Subsequently, libraries were quantified with the *Qubit™ HS DNA assay* (ThermoFisher Scientific, #Q32855), and library profiles were verified using the *DNA High sensitivity LabChip kit* using the Agilent 2100 Bioanalyzer (Agilent Technologies). Sequencing was carried out on an Illumina Nextseq 500 sequencer, generating 75-base paired-end reads. Quality control of the raw data was conducted using the *AOZAN* software, with the FastQC module (version 0.11.9). Reads were aligned to the mouse genome (GRCm38p6) using the *STAR* software [11] (RRID: SCR_004463; version 2.7.6a). Transcript quantification was performed with the *RSEM* software [12] (RRID: SCR_013027; version 1.3.1). Differential gene expression analyses were conducted using the R package DESeq2. And *p-values* were adjusted using the Benjamini-Hochberg method.

### 2.10 Glucose stimulated insulin secretion (GSIS)

KREBS buffer was prepared extemporaneously: 137 mM NaCl (VWR Chemicals, #27788.297), 5.36 mM KCl (Merck, #104933), 2.5 mM CaCl_2_ (Sigma–Aldrich, #C7902-500G), 0.81 mM MgSO_4_ (Sigma–Aldrich, #M7506-1 KG), 0.34 mM NaH_2_PO_4_ (Merck, #106346), 0.44 mM KH_2_PO_4_ (Sigma–Aldrich, #P5379-100G), 4.17 mM NaHCO_3_ (Sigma–Aldrich, #S5761-500G), 10 mM HEPES (Gibco, #15630056) and 0.4% BSA (Roche; #10775835001). The KREBS buffer pH was adjusted to pH 7.2–7.4. Mouse pancreatic islets or pseudo-islets (30–50) were primed in KREBS buffer without glucose for 1h at 37 °C, 5%CO_2_. Samples were then successively transferred into wells containing KREBS buffer containing (1) 2.8 mM, (2) 16.7 mM, (3) 2.mM glucose (Sigma–Aldrich, #G7528) and (4) 2.8 mM glucose + 50 mM KCl. Each incubation was carried out for 1h at 37 °C, 5%CO_2_. Supernatant was collected and stored at −20 °C until analysis for insulin by ELISA assay. For insulin content, islets cells were transferred in RIPA buffer (Sigma–Aldrich, #R0278-500 ML) supplemented with protease and phosphatase inhibitors (Complete Tablets, Roche #04693159001; PhosphoSTOP, Roche #04906837001). Insulin secretions and contents were measured using the Ultra-Sensitive Mouse ELISA kit (ChrystalChem, #90080), following the manufacturer's protocol and using the Tecan Spark 10M plate reader, and insulin quantities were calculated using a 4 parameters polynomial curve.

### 2.11 Dyn A measurement

For measurement of Dyn A content, groups of 30 pseudo-islets or pseudo-beta were transferred in RIPA buffer supplemented with protease and phosphatase inhibitors. Measures were performed using the Dynorphin A EIA kit (Phoenix Pharmaceuticals, #EK-021-03), following the manufacturer's protocol and using the Tecan Spark 10M plate reader.

### 2.12 cAMP level measurement

Islet clusters were dissociation with Accutase and resupended in HBSS^−/-^ (Life Technologies, #14170–088) supplemented with 10% FCS and 1% pen/strep. Subsequently, cAMP level measurements were performed using the HTRF cAMP kit (Revvity, #62AM4PEB). Cells were washed once with 1X PBS (ThermoFischer Scientific, #14190–094) supplemented with 1 mM IBMX (Sigma–Aldrich, #I7018-100 MG). Approximately 10,000 cells per well were dispensed into 384-well plate for HTRF (OptiPlate™-384, #6007290) and treated with 1 μM of FSK (forskolin, Tocris, #1099) in PBS/IBMX for 30 min at RT. Cells were next lysed for 1h at RT, and cAMP levels were measured according to the manufacturer's protocol. The plate was read using the Infinite F500 Tecan microplate reader, and cAMP levels were calculated using a non-linear regression curve.

### 2.13 Clusters of human beta-cell line

EndoC-βH1 [13] cells were cultured in Advanced DMEM F12 (Life Technology, #12634–010) with 2 mM GluatMAX (Life Technology, #35050–038), 2% BSA fraction V (Roche; #10775835001), 50 μM 2-mercaptoethanol (Sigma–Aldrich, #M3148), 10 mM nicotinamide (Alpha Aesar, #A12970), 6.7 ng/mL sodium selenite (Sigma–Aldrich, #S1382), penicillin/streptomycin. Cells were reaggregated in agarose molds for 1 day before being fixed and used for immunostaining.

### 2.14 Statistical analysis

For each experiment, “n" represents an independent biological sample. Statistical analyses were performed using Prism 8.0.2 software (GraphPad Software, San Diego, CA, USA). For qRT-PCR we used the *Mann–Whitney test* (for two groups comparison) or *One way ANOVA test* (for more than 2 groups comparisons), charts are presented as the median with the interquartile range (IQR). For Western Blot band quantification we used *paired t-test*, chart are presented as median with IQR. For insulin secretion and insulin content measurements we used the *Mann–Whitney test* (for two groups comparison) or the *multiple t-test* (for more than 2 groups comparisons), charts are presented as the mean with standard deviation (SD). For cAMP measurement we used the *Mann–Whitney test*, charts are presented as the mean with standard deviation (SD). A *p-value* below 0.05 was considered statistically significant. In the graphs, *p-values* are symbolized as following: ∗*P* < 0.05; ∗∗*P* < 0.01; ∗∗∗*P* < 0.005; ∗∗∗∗*P* < 0.001 and “ns” (not significant) *P* > 0.05.

### 2.15 Deposited data

The data from the RNAseq performed on beta-cells from pseudo-islets and pseudo-beta are available at the NCBI Gene Expression Omnibus (GEO): GSE292158.

https://www.ncbi.nlm.nih.gov/geo/query/acc.cgi?acc=GSE292158.

## 3 Results

### 3.1 Construction of chimeric mouse islet clusters

**Figure 1:**
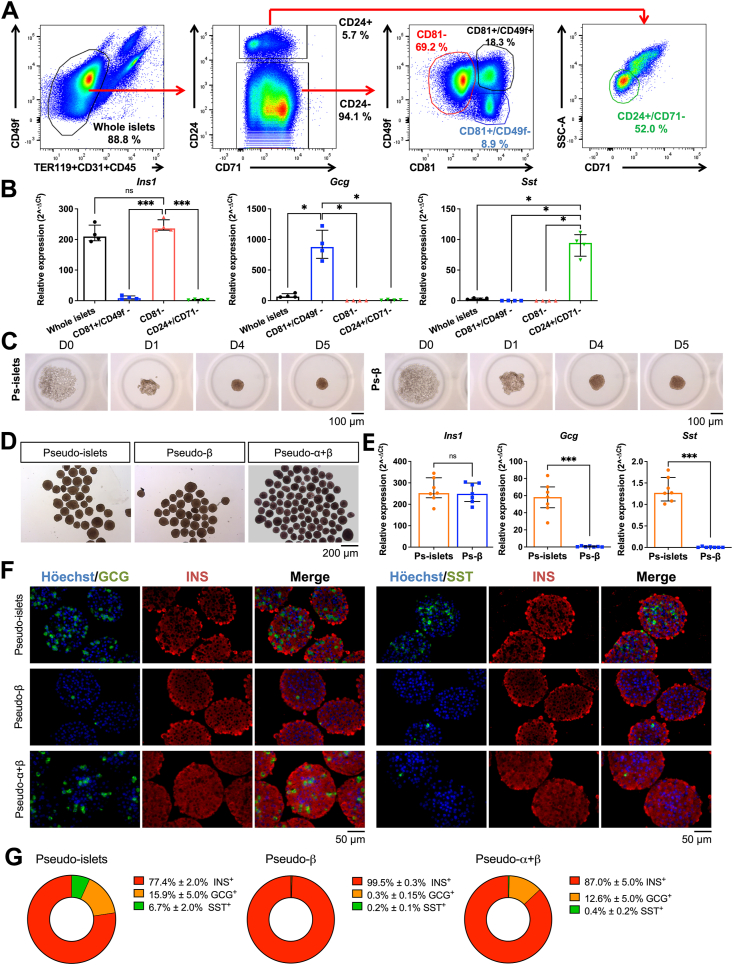
**Construction of chimeric mouse islet clusters. (A)** Representative flow cytometry plots of dispersed islets from adult C57BL/6 mice. CD24, CD71, CD49f and CD81 expression has been analyzed on endocrine cells after exclusion of dead cells (propidium iodide-positive cells) and hematopoietic and endothelial cells (CD45-, TER119-or CD31 positive cells) (left panel). Endocrine cells could be divided into CD24^high^ and CD24^low^ cells (middle panel). The CD24^low^ fraction could be further subdivided into three populations: CD81^-^; CD81^+^/CD49f^+^ and CD81^+^/CD49f^−^, while the CD24^high^ fraction comprise SSC-A^-^/CD71^-^ cells (right panels). Data are representative at least 90 flow cytometry experiments. **(B)** qRT-PCR analysis of *Ins1*, *Gcg* and *Sst* expression in whole islets versus sorted islet cell fractions (CD81^+^/CD49f^−^ in blue, CD81^-^ in red and CD24+/CD71-in green). From n = 4 independent sorting. ∗*P* < 0.05; ∗∗∗*P* < 0.005. **(C)** Brightfield pictures of pseudo-islets (left) and pseudo-β (right) cells at 2000 cells/clusters during the reaggregation process. Data are representative at least 80 independent experiments. **(D)** Brightfield pictures of pseudo-islets (left panel), pseudo-β (middle panel) or pseudo-α+β (right panel) after 5 days of reaggregation in culture. Data are representative of at least 3 independent experiments. **(E)** qRT-PCR analysis of *Ins1*, *Gcg* and *Sst* expression in pseudo-islets and pseudo-β after 5 days of culture. From n = 7 independent sorting. ∗∗∗*P* < 0.005. **(F, G)** Immunofluorescence staining on sections of pseudo-islets, pseudo-β and pseudo-α+β after 5 days of culture. Left panel: GCG in green, INS in red and nuclei in blue; right panel: SST in green, INS in red and nuclei in blue. The frequency of each cell type was quantified by measuring the area of INS^+^, GCG^+^ or SST^+^ in pseudo-islets, pseudo-β and pseudo- α+β from 3 independent experiments.

We previously developed a FACS-based method to simultaneously purify alpha-, beta- and delta-cell populations from young, adult and old mice [8,9]. In the current study, we first aimed to improve and facilitate this process by further enhancing alpha- and beta-cells distinction. For that purpose, we incorporated into our previous antibody panel (CD24, CD49f and CD71) CD81 as an alpha-cell marker [10]. We analyzed by flow cytometry dispersed islet cells from 12-week-old C57bl/6 mice, after excluding dead cells and non-pancreatic lineages (TER119^+^, CD31^+^ and CD45^+^). Through this approach, we observed four distinct populations: CD49f^+^CD81^-^ (69.2 ± 5%), CD49f^−^CD81^+^ (8.9 ± 4%), CD24^+^CD71^low^ (2.9 ± 1.5%) and CD49f^+^CD81^+^ (18.6 ± 5%) (Figure 1A). Using qRT-PCR, we specifically detected *Ins1* in CD49f ^+^ CD81^-^, *Gcg* in CD49f^−^CD81^+^ and *Sst* in CD24^+^ populations (Figure 1B). Moreover, comparative qRT-PCR with whole islets indicated strong enrichment for *Gcg* and for *Sst* in CD49f^−^CD81^+^ and in CD24^+^ populations respectively (Figure 1B).

**Supplementary Figure 1:**
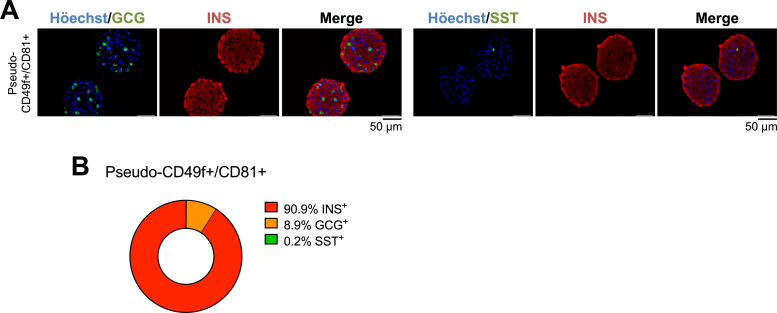
**(A**) Immunofluorescence staining on sections of islet clusters constructed with CD49f^+^CD81^+^ cell population following 5 days of culture. Left panel: GCG in green, INS in red and nuclei in blue; right panel: SST in green, INS in red and nuclei in blue. **(B)** Quantification of each cell type frequency by measuring the area of INS^+^, GCG^+^ and SST^+^ in islet clusters from CD49f^+^CD81^+^ population.

We next developed a protocol to reaggregate sorted cells into clusters. Mixed (alpha + beta + delta or alpha + beta) or beta only cell populations were loaded into microwell plates. During the 5 days of reaggregation, clusters became rounder and denser, and by the end of this process, aggregates were well-formed and easy to manipulate (Figure 1C), similar to native mouse islets. Clusters exhibited a homogenous spherical shape and size (approximately 100 μm), independent of their cell type composition (Figure 1D). Hereafter, we refer to as: pseudo-islets or pseudo-alpha + beta when cell clusters are composed of alpha-, beta- and delta-cells or alpha- and beta-cells respectively; and pseudo-beta for aggregates only composed of beta-cells. qRT-PCR analyses confirmed that pseudo-islets (Ps-islets) express *Ins1*, *Gcg* and *Sst* whereas pseudo-beta (Ps-β) express *Ins1* (Figure 1E). Immunofluorescence staining on sections of the different cluster types further confirmed their cell identities: Pseudo-islets contain alpha-, beta- and delta-cells in the same proportion as mouse pancreatic islets (77.4 ± 2% INS^+^, 15.9 ± 5% GCG^+^ and 6.7 ± 2% SST^+^ cells). Pseudo-beta clusters demonstrate high purity (99.5 ± 0.3% INS^+^, 0.3 ± 0.15% GCG^+^ and 0.2 ± 0.1% SST^+^ cells), while delta-cell-depleted pseudo-islets (Ps-α+β) were composed of alpha- and beta-cells (87.0 ± 5% INS^+^, 12.6 ± 5% GCG^+^ cells) with extremely rare SST^+^ cells (0.4 ± 0.2%) (Figure 1F,G for quantification). Note that the CD49f^+^CD81^+^ population (Figure 1A) contains a mix of INS^+^ and GCG^+^ cells (Suppl Fig. 1) and was not further analyzed in this study.

All together, we developed and validated a model of chimeric islets with tightly controlled proportions of alpha-, beta- and and-delta cells on demand, providing a robust tool for studying interactions among the three major cell populations that compose mouse pancreatic islets. With this innovative model, we investigated the role of alpha-/delta-cells on i). beta-cell sensitivity to glucose; ii). gene expression in beta-cells; and iii). beta-cell function.

### 3.2 Beta-cell sensitivity to glucose is independent of alpha-/delta-cells

**Figure 2:**
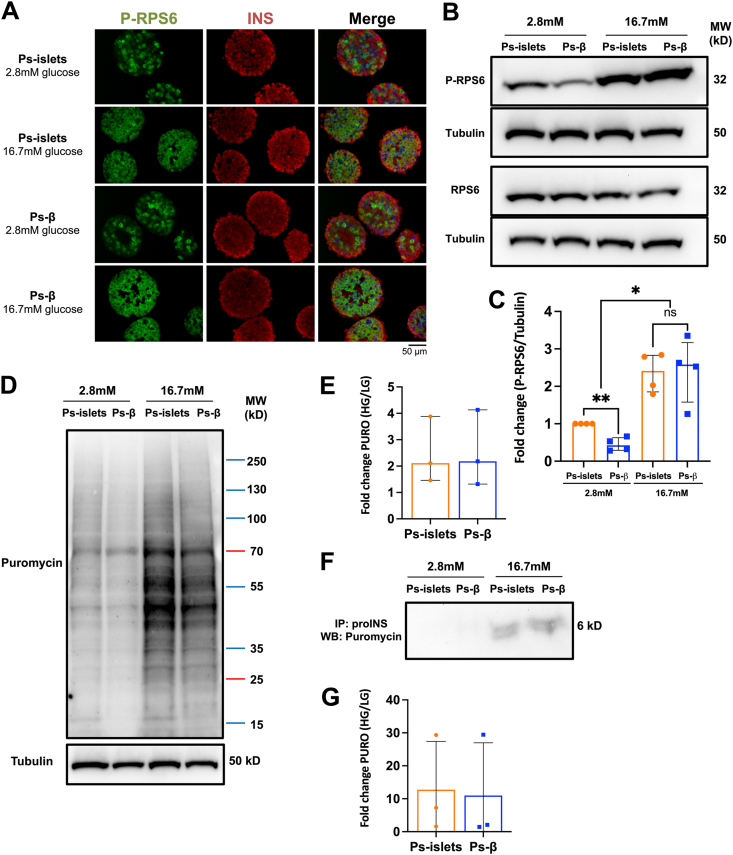
**Beta-cell sensitivity to glucose is independent of alpha-/delta-cells. (A)** Immunofluorescence staining on sections of pseudo-islets and pseudo-β stimulated with 2 mM or 16.7 mM of glucose (P-RPS6 in green, INS in red and nuclei in blue). **(B, C)** Western blot analysis and quantification of P-RPS6, RPS6 in pseudo-islets vs pseudo-β stimulated with 2 mM or 16.7 mM glucose. Tubulin was used as a loading control. n = 4 independent experiments. ∗*P* < 0.05; ∗∗*P* < 0.01. **(D, E)** Western blot analysis and quantification of puromycin incorporation in pseudo-islets vs pseudo-β stimulated with 2 mM or 16.7 mM glucose. n = 3 independent experiments. **(F, G)** Western blot analysis and quantification of puromycin incorporation in proINS immunoprecipitated from pseudo-islets vs pseudo-β stimulated with 2 mM or 16.7 mM glucose. n = 3 independent experiments.

We compared glucose sensitivity in beta-cells clustered in presence or absence of alpha-/delta-cells first using as a marker RPS6 phosphorylation (P-RPS6), known to increase upon glucose stimulation [[14], [15], [16]]. P-RPS6 analyses by either immunostaining (Figure 2A) or Western blot (Figure 2B,C for quantification) confirmed that glucose induced RPS6 phosphorylation in INS + cells. It also demonstrated that this induction was independent of the presence of alpha-/delta-cells. In beta-cells, glucose is known to increase translation [17] and used here as a readout to assess protein translation in our model. We observed that glucose stimulated puromycin incorporation, similarly in presence or absence of alpha-/delta-cells (Figure 2D,E for quantification). As a third approach, we pulsed pseudo-islets and pseudo-beta with puromycin and analyzed by Western blot puromycin incorporated into pro-insulin. Again, we observed similar induction of pro-insulin synthesis by glucose in presence or absence of alpha-/delta-cells (Figure 2F,G for quantification). Taken together, such data indicate that beta-cell sensitivity to glucose is independent on the presence of alpha-/delta-cells.

### 3.3 Alpha-/delta-cells control the expression of a specific set of genes in beta-cells

**Figure 3:**
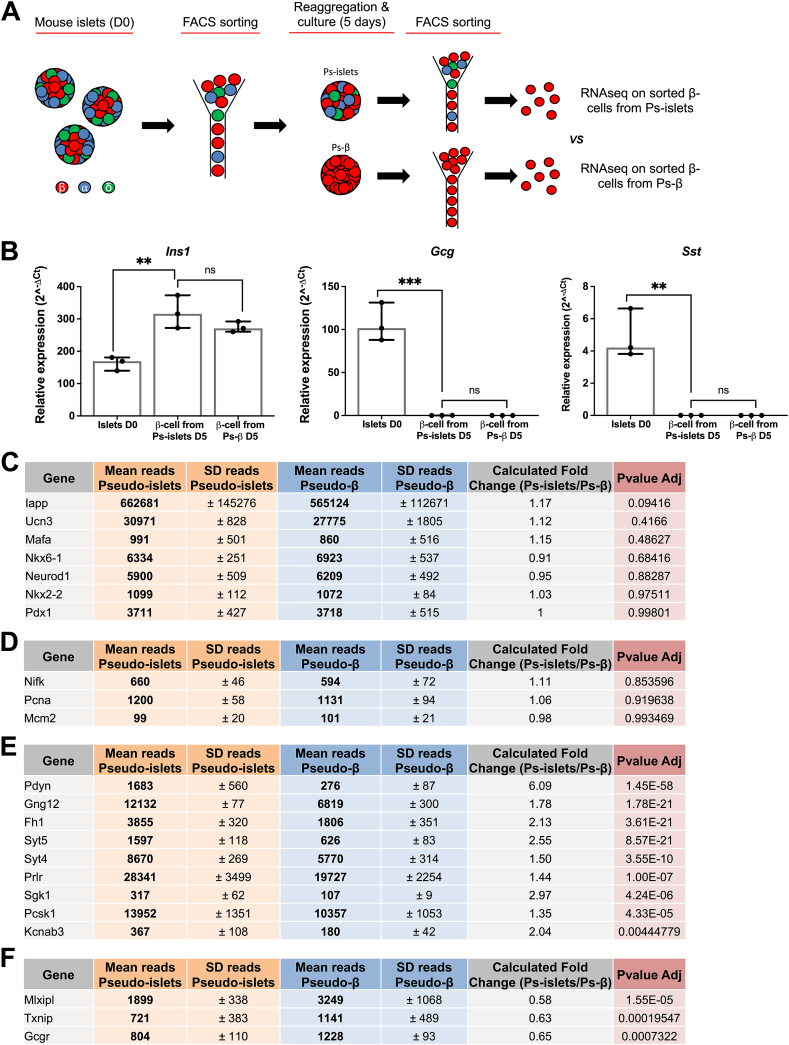
**Beta-cells proliferation and identity markers independence of alpha-/delta-cells signals. (A**) Schematic protocol used to determine the effect of signals from alpha-/delta-cells on beta-cells gene expression. **(B)** qRT-PCR analysis of *Ins1*, *Gcg* and *Sst* expression in beta-cells sorted from pseudo-islets and pseudo-β. n = 3 independent experiments. ∗∗*P* < 0.01; ∗∗∗*P* < 0.005. **(C, D)** RNAseq analyses of beta-cell identity **(C)** and proliferation **(D)** markers in beta-cells from pseudo-islets and pseudo-β. **(E, F)** Down- **(E)** and up **(F)** - regulated genes in beta-cells aggregated during 5 days of culture with or without alpha- and delta-cells. n = 3 independent experiments.

**Supplementary Figure 2:**
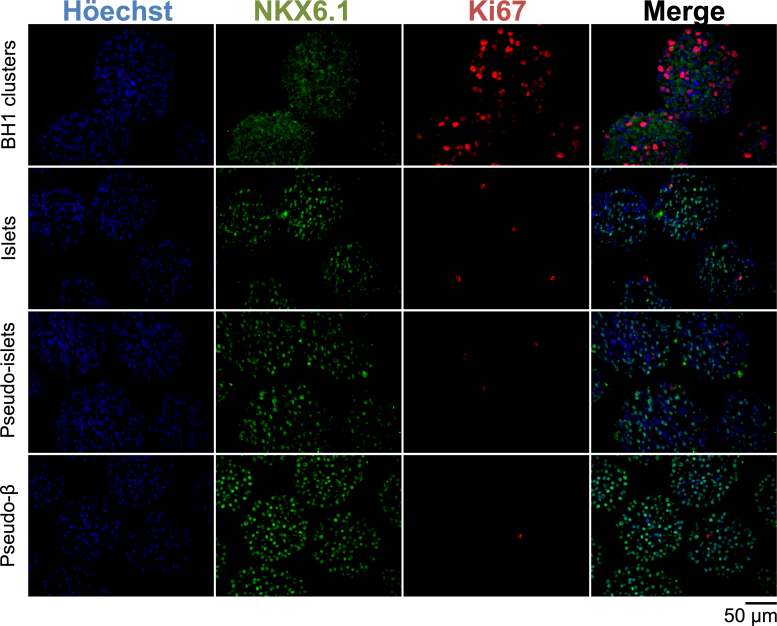
Immunofluorescence staining on sections of clustered BH1, mouse pancreatic islets, pseudo-islets and pseudo-β. NKX6.1 in green, Ki67 in red and nuclei in blue.

**Supplementary Figure 3:**
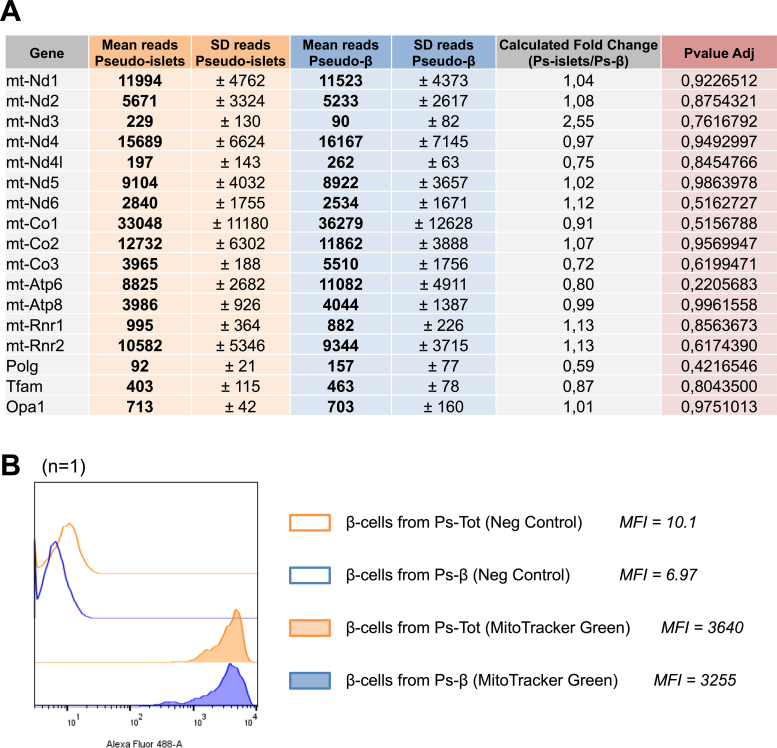
**(A)** RNAseq analyses of mitochondrial genes expression levels in beta-cells from pseudo-islets and pseudo-β. **(B)** FACS analysis of beta-cells from pseudo-islets and pseudo-β stained or not with MitoTraker Green.

We investigated whether signals derived from alpha-/delta-cells influence gene expression in beta-cells. To this end, we constructed islet clusters with or without alpha-/delta-cells, cultured them for 5 days and subsequently sorted beta-cells by FACS (Figure 3A). Quality control by qRT-PCR validated effective depletion of alpha- and delta-cells post-sorting (Figure 3B). RNAseq analysis indicated that canonical beta-cell markers (*Iapp*, *Ucn3, Mafa, Nkx6-1*, *NeuroD1, Nkx2.2*, and *Pdx1*) were similarly expressed following culture in presence or absence of alpha-/delta-cells (Figure 3C). It was also observed for beta-cell proliferation markers as measured by RNAseq (*Nifk*, *Pcna* and *Mcm2*) (Figure 3D) and by immunostaining for Ki67 (Suppl Fig. 2). Expression of mitochondrial genes assessed by RNAseq (Suppl Fig. 3A) and mitochondrial area measured by FACS using MitoTracker Green (Suppl Fig. 3B) were similar in beta-cells cultured with or without alpha-/delta-cells. Together, these results indicate that the absence of signals for alpha-/delta-cells does not alter beta-cell identity, proliferation or mitochondrial parameters.

**Supplementary Figure 4:**
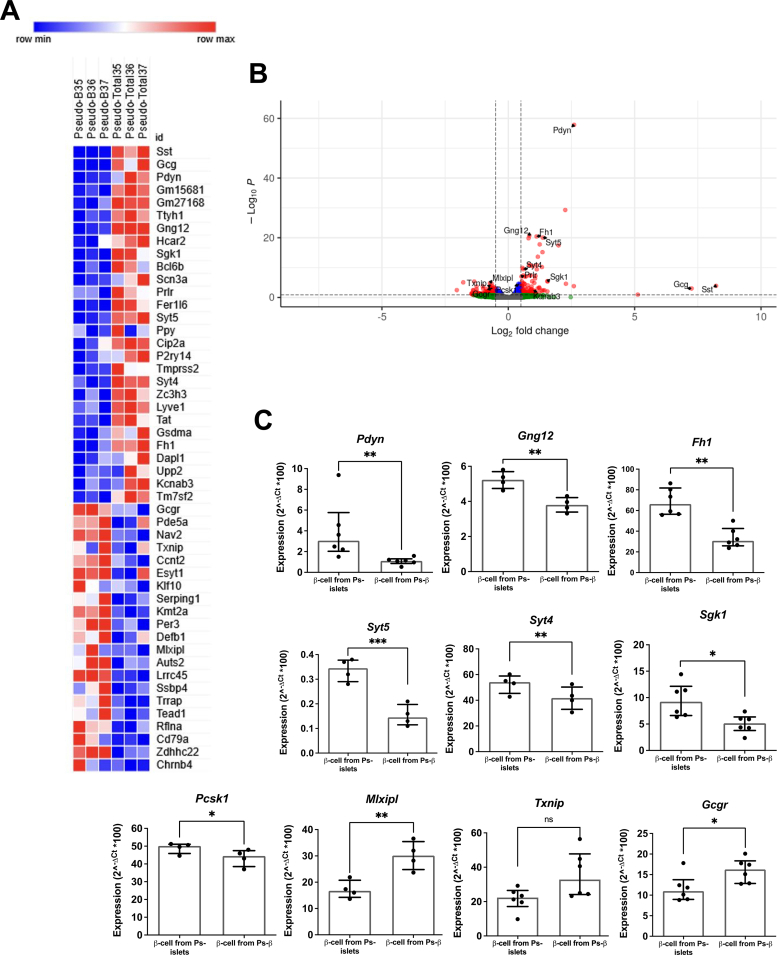
**(A)** Heat map from the RNAseq of the 49 most up- and down-regulated genes in beta-cells from pseudo-islets or pseudo-beta with a fold change exceeding 50% and an adjusted *Pvalue* below 0.05. **(B)** Volcano plot on RNAseq comparing gene expression in beta-cells from pseudo-islets and pseudo-beta. **(C)** qRT-PCR analysis of Pdyn, Gng12, Fh1, Syt5, Syt4, Sgk1, Pcsk1, Mlxipl, Txnip and Gcgr expression in sorted beta-cells from pseudo-islets and pseudo-β after 5 days of culture, n = 4–6 independent experiments. *∗P* < 0.05; *∗∗P* < 0.01; *∗∗∗P* < 0.005.

**Supplementary Figure 5:**
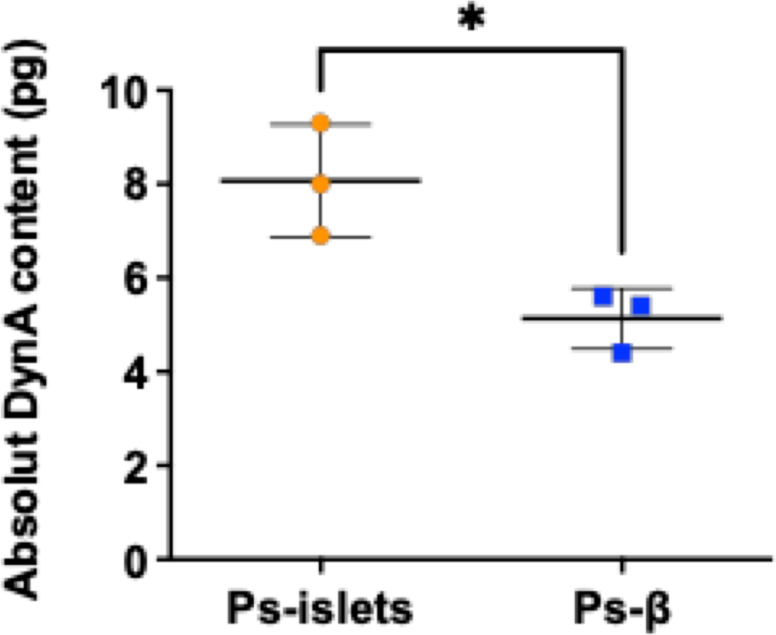
Quantification by ELISA of Dyn A content of pseudo-islets and pseudo-beta (n = 3 independent experiments). *∗P* < 0.05.

Interestingly, further data analysis revealed a list of 49 genes with a fold change greater than 50% (either up- or down-regulated) and with an adjusted *Pvalue* below 0.05 (Suppl Fig. 4A–B). In that list, we concentrated our effort on genes known or hypothesized to influence beta-cell function and selected 12 genes with the highest differential expression, excluding those predominantly expressed in alpha- or delta-cells to minimize the risk of contamination artifacts in pseudo-islets. Some genes showed decreased expression in beta-cells cultured in absence of alpha-/delta-cells (*Pdyn*, *Gng12, Fh1*, *Syt5, Syt4*, *Prlr*, *Sgk1*, *Pcsk1, Kcnab3*) (Figure 3E), while other exhibited increased expression (*Mlxipl, Txnip*, *Gcgr*) (Figure 3F). qRT-PCR analysis of independent samples further validated a unique set of beta-cell genes whose expression is regulated by alpha-/delta-cell signals (Suppl Fig. 4C). We further validated the decrease in PDYN expression in beta-cells cultured without alpha-/delta-cells at the protein level by ELISA (Suppl Fig. 5).

**Figure 4:**
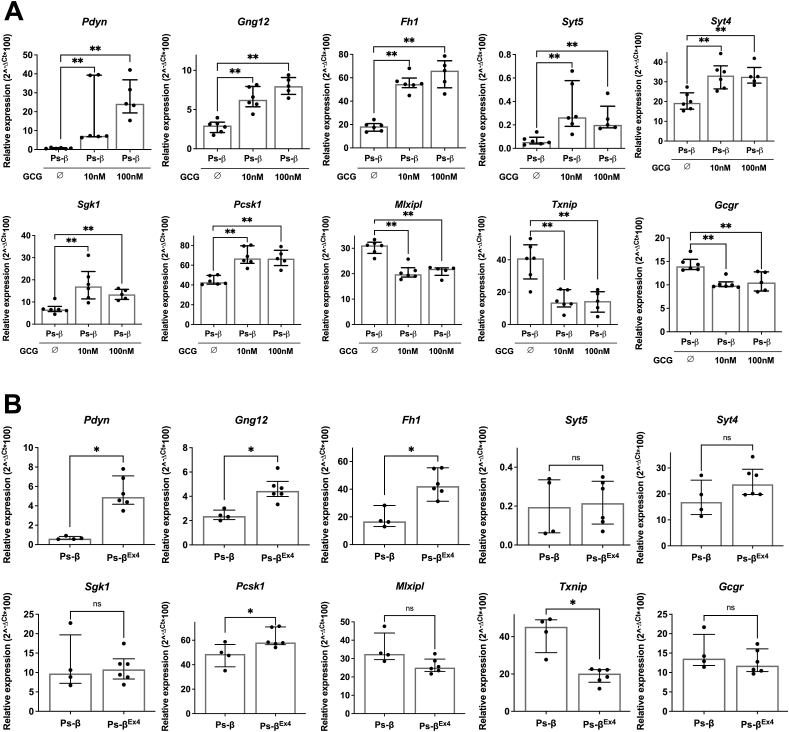
**Alpha-/delta-cells control the expression of a specific set of genes in beta-cells.** qRT-PCR analysis of *Pdyn*, *Gng12*, *Fh1*, *Syt5*, *Syt4*, *Sgk1*, *Pcsk1*, *Mlxipl*, *Txnip* and *Gcgr* expression in pseudo-β treated or not with 10 nM or 100 nM of GCG **(A)** or 1 nM of Ex4 **(B)** all over the reaggregation process (5 days), n = 3–6 independent experiments. ∗*P* < 0.05; ∗∗*P* < 0.01.

**Supplementary Figure 6:**
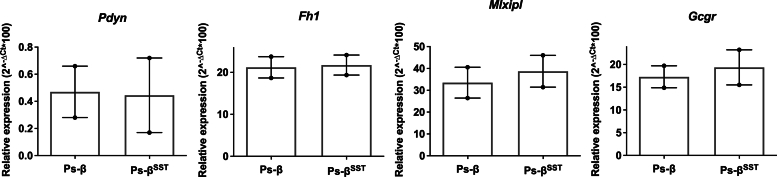
qRT-PCR analysis of *Pdyn*, *Fh1*, *Mlxipl* and *Gcgr* expression in pseudo-β and pseudo-β pre-treated with 200 nM of SST after 5 days of culture, n = 2 independent experiments.

We next investigated whether the observed effects of alpha-/delta-cells on beta-cell gene expression could be mimicked by glucagon (GCG). Treatment of beta-cell clusters with GCG during the 5-days reaggregation period increased the expression of *Pdyn*, *Gng12*, *Fh1*, *Syt5, Syt4, Sgk1* and *Pcsk1* (Figure 4A), while decreasing the expression of *Mlxipl, Txnip* and *Gcgr* (Figure 4A). Similarly, treatment with Exendin-4 (Ex4) that acts through the GLP1 receptor (GLP1R) increased the expression of *Pdyn*, *Gng12, Fh1, Pcsk1* and decreased *Txnip* expression (Figure 4B). Note that treatment of pseudo-beta with SST did not influence the expression levels of *Pdyn*, *Fh1*, *Mlxipl* or *Gcgr* (Suppl Fig. 6). Taken together, we demonstrate that signals from alpha-cells regulate the expression of a new specific set of genes in beta-cells through the GCG receptor (GCGR) and GLP1R pathways.

### 3.4 Insulin secretion is dependent of signals from alpha-cells

**Figure 5:**
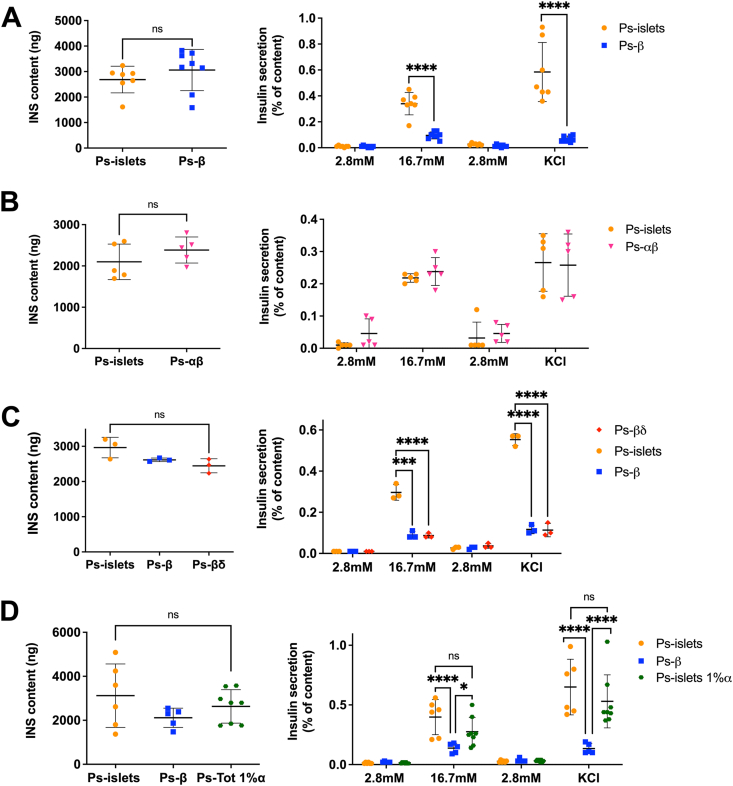
**Insulin secretion is dependent of signals from alpha-cells.** Insulin content (left panels) and glucose- and KCl-stimulated insulin secretion (right panels) of pseudo-islets vs **(A)** pseudo-β (n = 7–8 independent experiments), **(B)** pseudo-β+α (n = 5 independent experiments), **(C)** pseudo-β+δ (n = 3 independent experiments) and **(D)** pseudo-islets 1% α-cells (n = 3–4 independent experiments). ∗∗∗*P* < 0.005; ∗∗∗∗*P* < 0.001.

We analyzed beta-cell function in islet cell clusters depleted or not of alpha-/delta-cells. There, we tested the effects of glucose and KCl on insulin secretion with a semi dynamic protocol: glucose was applied sequentially 2.8 mM, 16.7 mM, a return to 2.8 mM (baseline), followed by stimulation with KCl (50 mM). Total insulin content remained unchanged regardless of the presence or absence of alpha-/delta-cells (Figure 5A, left), while insulin secretion was altered. In pseudo-islets, glucose (16.7 mM) robustly stimulated insulin secretion, which returned to baseline at 2.8 mM and was further stimulated by KCl. On the other hand, in the absence of alpha- and delta-cells, response to both glucose and KCl was significantly blunted (Figure 5A). To distinguish the role of alpha-versus delta-cells in this effect, we selectively removed one of the cell types. We observed that removing delta-cells did not impact glucose- or KCl-stimulated insulin secretion (Figure 5B). In contrast, removing alpha-cells significantly blunted insulin secretion in response to both glucose and KCl, mimicking the phenotype observed with pseudo-beta (Figure 5C). Note that insulin content was not modified in any of the conditions tested, which matches with the lack of effect of alpha-/delta-cells on insulin gene expression (Figure 3C) and protein translation (Figure 2D–G) in beta-cells. We next asked whether a limited number of alpha-cells would be sufficient to restore sensitivity to glucose and KCl. We constructed pseudo-islets with only 1% of alpha-cells (compared to ±15% in control pseudo-islets) and compared these with pseudo-beta. We observed that this limited number of alpha-cells was sufficient to restore glucose- and KCl-induced insulin secretion (Figure 5D). We concluded that alpha-cells (but not delta-cells) are essential for efficient glucose- or KCl-stimulated insulin secretion.

### 3.5 Activators of the cAMP pathway and calcium channel openers, but not potassium channel inhibitors, restore insulin secretion in the absence of alpha-/delta-cells

**Figure 6:**
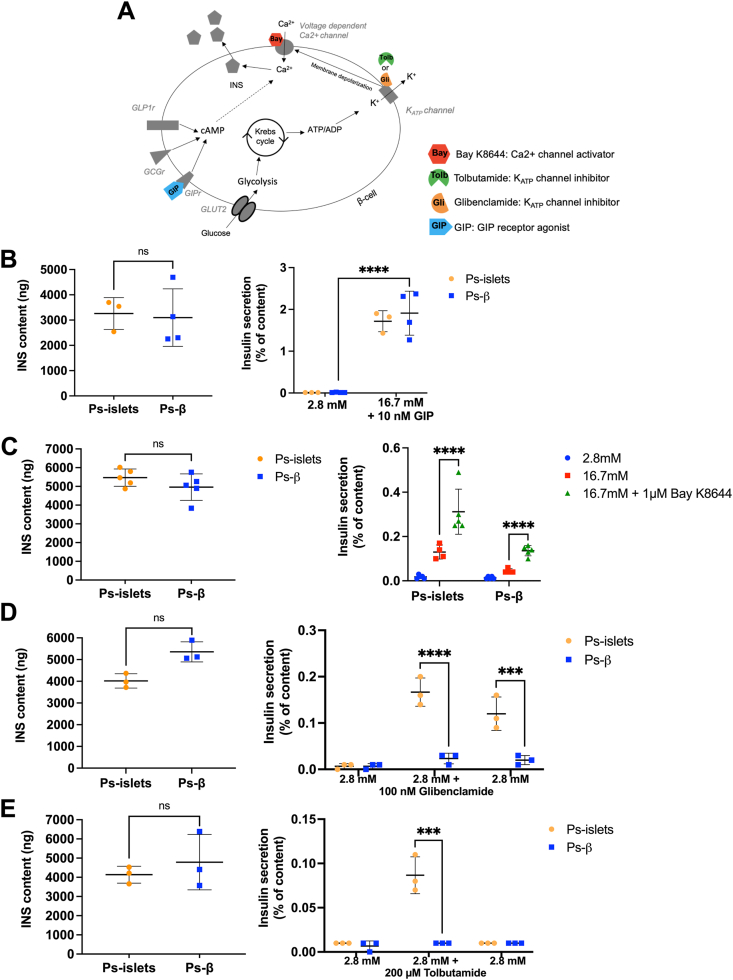
**Activators of the cAMP pathway and calcium channel openers, but not potassium channel inhibitors, restore insulin secretion in the absence of alpha-/delta-cells. (A)** Beta-cell scheme with modulators of insulin secretion that we used. **(B**–**E)** Effect of GIP **(B)**, Bay K8644 **(C)**, Glibenclamide **(D)** and Tolbutamide **(E)** on insulin content (left panels) and glucose-stimulated insulin secretion (right panels) from pseudo-islets vs pseudo-β. n = 3–5 independent experiments. ∗∗∗*P* < 0.005; ∗∗∗∗*P* < 0.001.

We used activators and inhibitors to elucidate the mechanism underlying the lack of insulin secretion in pseudo-beta upon glucose stimulation (Figure 6A). We observed that in the presence of GIP, which increases cAMP levels, or Bay K8644, a calcium channel opener, glucose successfully induced insulin secretion in pseudo-beta (Figure 6B–C). On the other hand, glibenclamide and tolbutamide, 2 sulfonylureas that close the ATP-sensitive potassium (K_ATP_) channel, induce insulin secretion in whole islets but not in pseudo-beta (Figure 6D–E). All together, these results indicate that in the absence of alpha-/delta-cells, impaired glucose-stimulated insulin secretion is due to a K_ATP_ channel default.

### 3.6 Pseudo-beta pretreated with GLP1 regain glucose-stimulated insulin secretion

**Figure 7:**
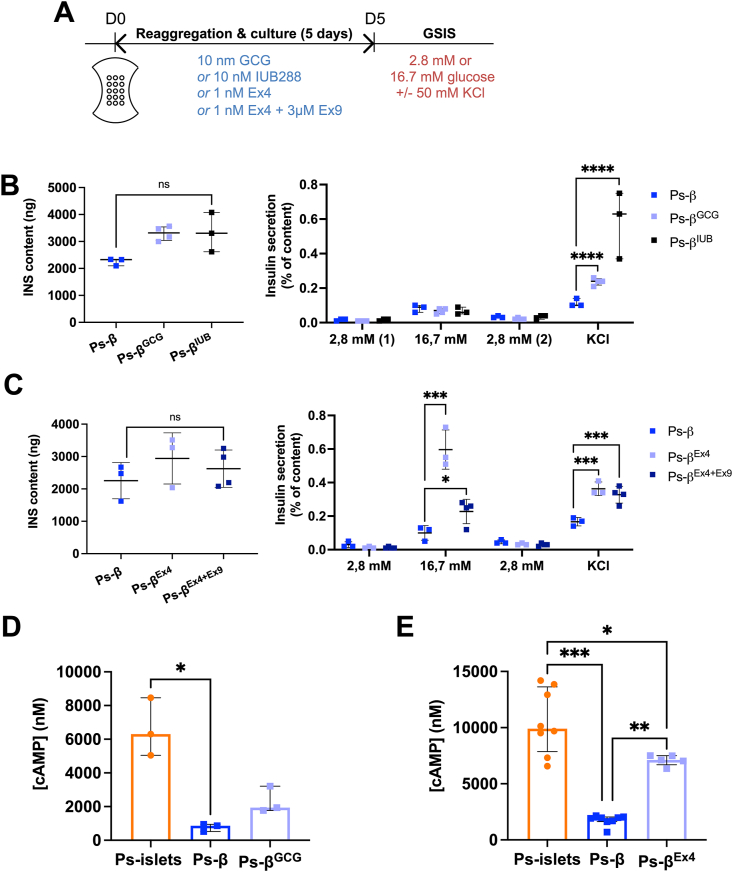
**Pseudo-beta pretreated with GLP1 regain glucose-stimulated insulin secretion. (A**) Experimental scheme for pseudo-islets and pseudo-β pre-treatment with 10 nm GCG, 10 nM IUB288, 1 nM Ex4 or 1 nM Ex4 + 3 μM Ex9. **(B, C)** Effect of a 5-days pre-treatment during the reaggregation period with GCG, IUB288 **(B)**, Ex4, Ex4 + Ex9 **(C)** on insulin content (left panels) and glucose- and KCl-stimulated insulin secretion (right panels) from pseudo-β. n = 3–4 independent experiments. ∗∗∗*P* < 0.005; ∗∗∗∗*P* < 0.001. **(D, E)** Measurement of cAMP levels on pseudo-islets, pseudo-β, pseudo-β pre-treated with 10 nM GCG **(D)** and pseudo-β pre-treated with 1 nM Ex4 **(E)**. n = 3–8 independent experiments. ∗*P* < 0.05; ∗∗*P* < 0.01; ∗∗∗*P* < 0.005; ∗∗∗∗*P* < 0.001.

**Supplementary Figure 7:**
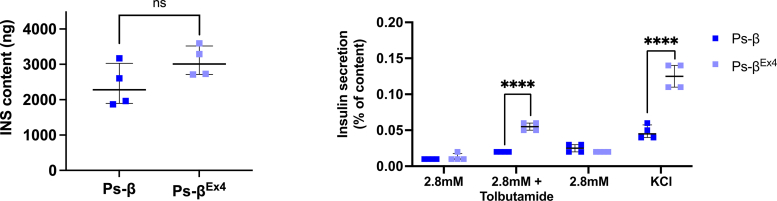
Effect of a 5 days pretreatment with Ex4 on Tolbutamide-stimulated insulin secretion from pseudo-β. n = 4 independent experiments. *∗∗∗∗P* < 0.001.

To assess whether GCG could restore insulin secretion, we added GCG during the 5-days reaggregation process (Figure 7A). We observed that this pre-treatment rescued KCl-induced but not glucose-induced INS secretion (Figure 7B). Similar results were obtained when GCG was replaced by IUB288, a potent GCGR agonist, during the reaggregation period (Figure 7B). We next tested Ex4 during the reaggregation period and observed that this pre-treatment restored KCl- and glucose-stimulated insulin secretion (Figure 7C). Importantly glucose-induced insulin secretion was sharply reduced when pseudo-beta were treated with Ex4 plus Exendin (9–39), a GLP1R antagonist [18] during the 5-days reaggregation period (Figure 7C). Interestingly, Ex4 during the reaggregation period also restored tolbutamide-induced insulin secretion (Suppl Fig. 7). Pre-treatment of pseudo-beta with SST during the reaggregation period had no impact on the insulin secretion capacity is these clusters (data not shown). Taken together, our data indicate that activation of the GLP1R and GCGR in beta-cells are respectively necessary for efficient glucose-and KCl-induced insulin secretion.

Measurement of cAMP levels in clusters with or without alpha-/delta-cells revealed that the amount of cAMP was considerably lower in pseudo-beta compared to pseudo-islets (Figure 7D–E). Pre-treatment of pseudo-beta with GCG did not improve cAMP levels (Figure 7D) while pre-treatment with Ex4 increases cAMP production compared to untreated pseudo-beta (Figure 7E). These results suggest that the rescue of glucose-stimulated insulin secretion observed in pseudo-beta pre-treated with GLP1R agonist is due to an enhanced capacity for cAMP production. This demonstrates that beta-cells require constant stimulation through the GLP1R and need a high capacity for cAMP production to display proper glucose-stimulated insulin secretion.

## 4 Discussion

In this study, we have developed a method to purify by FACS mouse alpha-, beta- and delta-cell populations, followed by their reaggregation to give rise to reconstructed chimeric mouse islets. Using this simple and innovative approach, we achieved the following: (i) we can generate on demand clusters with different proportions of alpha, beta- and delta-cells; (ii) we found that beta-cell glucose sensitivity is independent of the presence of alpha- and delta-cells; (iii) we discovered in beta-cells a subset of genes regulated through alpha-cell signaling via GCGR and GLP1R; and (iv) we found that beta-cell function is dependent of alpha-cell signaling through GCGR for KCl-induced beta-cell activity and GLP1R for glucose-induced insulin secretion.

We previously developed a FACS-based approach to prepare alpha-, beta- and delta-cell populations. The protocol was based on positive selection of beta- and delta-cells using the cell surface markers CD71 and CD24, while alpha-cells were negatively sorted. This method gave rise to some contaminations between alpha- and beta-cell populations [8,9]. Recently, CD81, a tetraspanin surface protein involved in membrane organization, protein trafficking, cellular fusion and cell–cell interactions [19,20] was identified as being expressed by mouse islets and enriched in alpha-cells compared to beta-cells. [[8], [9], [10]]. By incorporating CD81 into our antibody panel for cell sorting, we enhanced the distinction between alpha- and beta-cells, thereby reducing beta-cell contamination by alpha-cells to less than 1% (Figure 1G).

In beta-cells, glucose plays major roles not only for insulin secretion, but also for protein translation [17]. As an example, glucose activates the mTOR pathway [21] and increases biosynthesis of major beta-cell proteins such as pro-insulin [22] and islet amyloid pancreatic polypeptide [23]. However, whether signals from alpha- and delta-cells regulate protein translation in beta-cells remains poorly studied. Here, we observed that glucose activates the mTOR pathway (measured by RPS6 phosphorylation) and protein synthesis (measured by puromycin incorporation into newly-synthesized proteins including pro-insulin) at similar levels in reconstructed chimeric islets depleted or not of alpha- and delta-cells. This indicates that the effect of glucose on protein synthesis is beta-cell autonomous and independent on signals from alpha- and delta-cells.

Interestingly, by performing transcriptomic analyses of beta-cells cultured with or without alpha- and delta-cells, we discovered that: (i) genes encoding beta-cell identity, proliferation and mitochondrial proteins are not modulated by neighboring alpha- and delta-cells, indicating that while beta-cells can lose their differentiation status [24], this loss seems to be independent of signals from alpha- and delta-cells. (ii) A specific set of beta-cell genes is modulated by signals from neighboring endocrine cells. For example, the expression of *Pdyn* shows a sharp decrease in beta-cells from pseudo-beta, which was restored when these pseudo-beta islets were pretreated with GCG or Ex4, but not with SST. This indicates that *Pdyn* expression in beta-cells is depending on signals from alpha-cells. Past studies have shown that the opoid peptide Dyn A, a processed product of Pro-dynorphin, is secreted by beta-cells in response to glucose, similar to insulin [25] and can stimulate the κ-opioid receptor on alpha-cells, enhancing glucagon secretion [26]. Combining these data with ours suggests that Dyn A could be implicated in an alpha-beta cell positive feedback loop: glucagon would activate Dyn A production by beta-cells which would then act on alpha-cells to increase glucagon secretion in a glucose-dependent manner. Dyn A production could also serve as markers of impaired communication between alpha- and beta-cells and be used to determine when insulin secretion deficits are due to a lack of beta-cell stimulation by alpha-cells.

We observe a sharp decrease in glucose- and KCl-stimulated insulin secretion when beta-cells are cultured in the absence of alpha-cells. This aligns with a set of data indicating that insulin secretion depends on intra-islet glucagon signaling [5,6,27]. However, this seems to contradict a recent study indicating that non-beta-cells are not required for accurate insulin secretion [7]. Such differences could be explained by technical differences as in [7], the authors use islets where alpha-, delta- and PP-cells were destroyed by diphteria toxin following complex crosses between genetically modified mice. These differences could also be explained by the presence of a few remaining alpha-cells. Indeed, using our model of reconstructed chimeric islets, we observed that 1% alpha-cells is sufficient for proper glucose-stimulated insulin secretion. Difference could finally be due to a mechanism of *in vivo* priming of beta-cells by endogenous GCG or GLP1 produced by intestinal L-cells [28]: in the diphteria toxin model, plasma GCG levels are reduced by only 35% one week after treatment with the toxin [29]. Additionally, there is an increase in GLP1 plasma levels during oral glucose tolerance test compared to control mice [7]. Here, we demonstrated that pretreatment of pseudo-beta with GCG or GLP1 restores insulin secretion, which suggests that these signals in the diphteria toxin model might explain the differences between the two models.

Sulfonylureas, such as tolbutamide and glibenclamide, are known to stimulate insulin secretion by closing the K_ATP_ channel, thereby depolarizing the beta-cell membrane [30]**.** They have been used for decades in treatment for type 2 diabetes [31]. However, it has been reported that prolonged treatment with sulfonylureas can lead to failure, occurring at a rate of 5%–10% per year, which has been suggested to be due to beta-cell apoptosis [32]. In our experiments, insulin secretion stimulated by sulfonylurea is significantly decreased in pseudo-beta. Importantly, treating pseudo-beta with GCG throughout the reaggregation process perfectly restores sulfonylurea-stimulated insulin secretion. This demonstrates that alpha-cell communication via the GCG receptor signaling pathway is crucial for sulfonylureas-stimulated insulin secretion. Therefore, we propose that the resistance to sulfonylurea treatment observed over time in T2D patients could be due to insufficient stimulation of beta-cells through the GCG receptor (possibly due to reduced GCG secretion by alpha-cells) rather than beta-cell apoptosis.

The strength of the chimeric model we developed lies in its use of primary mouse islet cells. The technique is easy to use and applicable to various mouse strains. This model complements previous engineered mouse models with specific deletions of alpha- or delta-cells [33,34]. A strength of this model is its usefulness for studying the influence of type 2 diabetes (T2D) drugs (like sulfonylurea or GLP1 tested in the present study) on beta-cell function and determine whether these drugs directly impact beta-cell or first affect alpha- or delta-cells.

In conclusion, our innovative chimeric islet model provides key insights into the complex mechanisms governing beta-cell behavior within pancreatic islets.

## CRediT authorship contribution statement

**Alexis Fouque:** Writing – review & editing, Writing – original draft, Visualization, Validation, Methodology, Investigation, Formal analysis, Conceptualization. **Masaya Oshima:** Validation, Methodology, Investigation, Formal analysis, Conceptualization. **Nina Mode:** Validation, Methodology, Investigation. **Romain Ducellier:** Validation, Methodology, Investigation. **Delphine Thibaut:** Validation, Methodology, Investigation. **Florence Gbahou:** Validation, Resources, Methodology, Investigation. **Latif Rachdi:** Methodology, Conceptualization. **Over Cabrera:** Writing – review & editing, Conceptualization. **Raphaël Scharfmann:** Writing – review & editing, Writing – original draft, Visualization, Methodology, Funding acquisition, Conceptualization.

## Funding

This project has received funding from the European Union’s Horizon 2020 research and innovation programme under grant agreement no. 874839 (RS), l'Agence Nationale de la Recherche (ANR-21-CE14-0001-01) (RS), the Laboratoire d’Excellence consortium Revive (Investissements d'Avenir ANR-10-LABX-73-01) (RS) and from Lilly (RS and OC). AF is supported by PhD grant from Laboratoire d’Excellence consortium Revive.

## Declaration of competing interest

The authors declare that they have no known competing financial interests or personal relationships that could have appeared to influence the work reported in this paper.

## Data Availability

Data will be made available on request.
