# Supplementary material for: Constructing chimeric mouse islets to study alpha- and delta-cell influence on beta-cell feature
Source: Mol Metab. 2025 Sep 1;101:102245. doi: 10.1016/j.molmet.2025.102245 (PMC12544172; doi:10.1016/j.molmet.2025.102245)
Supplement: Multimedia component 1 [file mmc1.docx]

# **Supplementary Table**

## Supplementary Table 1:

List of primers used for qRT-PCR analysis

| Target gene | Forward seq (5’->3’) | Reverse seq (5’->3’) |
| --- | --- | --- |
| *Ppia* | CAGGTCCTGGCATCTTGTCC | TGCTTGCTGGTCTTGCCATTCC |
| *Ins* | CAGAGACCATCAGCAAGCAG | GGGACCACAAAGATGCTGTT |
| *Gcg* | TGAAGACAAACGCCACTCAC | TGACGTTTGGCAATGTTGTT |
| *Sst* | TCCGTCAGTTTCTGCAGAAGTCTC | GTACTTGGCCAGTTCCTGTTTCCC |
| *Pdyn* | CCTTCTGAATCTTGGATCGGC | GATCTCAAAGCCTGGGGATGA |
| *Sgk1* | TCCTATGCACCTCCTGTGGA | TTCTGTCAGCTGGCAACTCC |
| *Fh1* | AGCCAGAGCTCGAATGACAC | TGATGACCTGCGCAAACTCT |
| *Kcnab3* | TGGAACATCTGGGCTCCCTA | ACAATCACTCCGCATTGGGT |
| *Gcgr* | CAGGGCCTTGTCATGGTGAT | GAGATTCAGGTGGGGCTGTC |
| *Txnip* | CGAGTCAAAGCCGTCAGGAT | CGTTCTCACCTGCTGTAGGC |
| *Mlxipl* | AATGGGATGGTGTCTACCGC | GGCGAAGGGAATTCAGGACA |
| *Gng12* | AGGACTTTGGGGTGAGCATC | CTGAAAATCTTACCAGCAAGGC |
| *Pcsk1* | AGGTCGAGTCTAGCTGGTGT | TGCTCCATGGCTCAAAACCT |
| *Prlr* | TCTCATCTTGGCTACTGGGAGA | GCATGATCAGGGGACCTTCT |
| *Syt4* | TTTGTGTGCTTAGCCAGTCT | TGCTTCCCTCCGGATCACTA |
| *Syt5* | GTCCTGTCGAGACACCTAGCA | GCTTCCTGCAGAGGTACCCAAA |
